# The Moderating Effect of Focused Eating Between Body Dysmorphic Concerns and Eating Attitudes in Lebanese Dietitians

**DOI:** 10.1002/hsr2.72594

**Published:** 2026-06-04

**Authors:** Elie Ghadban, Joya Sassine, Sahar Obeid, Lucia Helou, Yonna Sacre, Nada Akiki, Feten Fekih‐Romdhane, Souheil Hallit, Marie Hokayem

**Affiliations:** ^1^ School of Medicine and Medical Sciences Holy Spirit University of Kaslik Jounieh Lebanon; ^2^ Department of Nutrition and Food Sciences, Faculty of Arts and Sciences Holy Spirit University of Kaslik (USEK) Jounieh Lebanon; ^3^ Department of Psychology and Education, School of Arts and Sciences Lebanese American University Jbeil Lebanon; ^4^ The Tunisian Center of Early Intervention in Psychosis, Department of Psychiatry “Ibn Omrane” Razi Hospital Manouba‐Tunisia Tunisia; ^5^ Faculty of Medicine of Tunis University of Tunis El Manar Tunis Tunisia; ^6^ Applied Science Research Center Applied Science Private University Amman Jordan

**Keywords:** body dysmorphic concerns, dietitians, focused eating, inappropriate eating attitudes, Lebanon

## Abstract

**Background:**

Body dysmorphic concerns are associated with unhealthy eating attitudes, but the role of focused eating in this relationship remains unclear. Given the rising prevalence of body image issues among young dietitians, it is essential to explore psychological factors that may help reduce disordered eating behaviors. This study aims to examine the moderating effect of focused eating on the association between body dysmorphic concerns and inappropriate eating attitudes among Lebanese dietitians.

**Methods:**

A cross‐sectional study was conducted among 215 Lebanese dietitians using a self‐administered questionnaire. The questionnaire included the Arabic validated versions of the Mindful‐Eating Behaviors Scale (MEBS), the Dysmorphic Concerns Questionnaire (DCQ), and the Eating Attitudes Test (EAT‐7). Moderation analysis was performed using the PROCESS MACRO v3.4 in SPSS, with adjustments for potential confounders such as body mass index, marital status, and employment status.

**Results:**

The findings revealed that focused eating moderated the relationship between body dysmorphic concerns and inappropriate eating attitudes (*β* = −0.03, *t* = −2.10, *p* = 0.037, 95% CI [−0.05, −0.002]). Higher body dysmorphic concerns were significantly associated with greater inappropriate eating attitudes at low levels (*β* = 0.32, *p* < 0.001) and moderate levels (*β* = 0.21, *p* < 0.001) of focused eating.

**Conclusion:**

These findings suggest that, within this sample of predominantly female young Lebanese dietitians, focused eating was associated with a weaker relationship between body dysmorphic concerns and inappropriate eating attitudes. Given the sampling approach and cross‐sectional design, these findings should be interpreted cautiously and cannot be generalized to all dietitians. Further longitudinal and interventional studies are needed before translating these findings into broader educational or healthcare initiatives.

## 1 Introduction

Body image concerns are increasingly challenging among young professionals in the healthcare sector, particularly dietitians, who are often perceived as embodying idealized standards of nutrition and health [1]. Body dysmorphic disorder (BDD) is characterized by an excessive preoccupation with perceived physical flaws, leading to significant psychological distress and functional impairments [1, 2]. Body dysmorphic concerns (BDC), on the other hand, describe milder, subclinical worries about appearance that, while not meeting diagnostic criteria for BDD, can similarly still result in excessive mirror‐checking, grooming, reassurance‐seeking, and appearance comparisons [3, 4, 5, 6]. Rather than forming a discrete disorder, body dysmorphic symptoms appear to vary continuously within the general population [7]. On this spectrum, body dysmorphic concerns may denote either a risk state for BDD or a subclinical expression of its cognitive‐emotional features [8, 9].

In addition to increasing vulnerability to BDD, the impact of subclinical body dysmorphic symptoms extends beyond mental well‐being, often contributing to comorbidities such as depression, social anxiety, obsessive‐compulsive disorder, compulsive sexual behavior, and disordered eating patterns, all of which can increase the risk of suicidal ideation and attempts [2, 4, 6, 10, 11, 12, 13, 14, 15]. Previous research suggests that individuals with severe body dysmorphic concerns often experience a reduced quality of life and engage in disordered eating behaviors such as restrictive dieting, binge eating, and an obsessive focus on food content and health [10, 16, 17, 18, 19]. They also exhibit a strong desire for cosmetic surgery, which may lead to surgical addiction and other adverse consequences [20, 21, 22]. Additionally, individuals with body dysmorphic concerns often experience increased body shame and fluctuations in self‐esteem, which may further impact their personal and professional lives [23, 24]. For dietitians, these challenges could potentially impair their ability to provide balanced nutritional guidance, as their personal insecurities may inadvertently influence their professional recommendations. To our knowledge, this issue has not been explicitly examined in the existing literature.

### 1.1 Body Dysmorphic Concerns, Disordered Eating, and Their Impact on Nutrition and Mental Health

Previous literature suggests that higher levels of body dysmorphic concerns can contribute to unhealthy eating attitudes, an excessive focus on healthy eating, and, in some cases, disordered eating behaviors [11, 25].

This preoccupation is often characterized by a persistent concern with dietary choices, particularly among young dietitians, who may spend significant amounts of time planning meals, inspecting nutrition labels, and second‐guessing their food selections [17]. Consequently, this intense preoccupation with food can disrupt professional responsibilities and hinder the promotion of balanced eating habits. The National Eating Disorders Association supports this claim, noting that dysregulated eating patterns contribute to reduced productivity in both diagnosed and undiagnosed individuals due to heightened emotional and mental strain [26].

Although disordered eating and clinically diagnosed eating disorders share similarities, they differ in diagnostic criteria and severity. Disordered eating encompasses a wide range of problematic food‐related behaviors and attitudes toward body image [27]. These behaviors may include meal skipping, restrictive dieting, food avoidance, binge eating, excessive exercise, or reliance on weight control methods such as diuretics, laxatives, and supplements [27, 28]. Unlike clinically diagnosed eating disorders, disordered eating does not always meet the established thresholds for frequency, duration, or psychological distress required for a formal diagnosis [27].

In contrast, eating disorders are formally classified under the Diagnostic and Statistical Manual of Mental Disorders (DSM‐5‐TR) as conditions characterized by persistent dysregulations in eating behaviors and significant psychological distress. These disorders include anorexia nervosa, bulimia nervosa, binge eating disorder, avoidant/restrictive food intake disorder (ARFID), and other specified eating disorders [29].

In Lebanon, healthcare professionals, particularly dietitians, are regarded as the most trusted sources of information on dietary habits and nutrition‐related concerns [30, 31]. A study conducted by Hoteit et al. in Lebanon revealed that females are at a higher risk of developing eating disorders compared to males, with nutrition students representing the most vulnerable group [32].

Moreover, it is essential to examine the body mass index (BMI) of participants as a key variable, since previous findings have established a connection between body dysmorphic concerns, BMI, and eating disorder symptomatology [33]. Research showed that 12% of individuals diagnosed with eating disorders also experienced coexisting body dysmorphic concerns, which were not necessarily associated with weight [11]. Instead, dissatisfaction was more commonly reported in relation to non‐weight‐related features, including hair, skin, nose, teeth, and height, with dissatisfaction levels ranging from 20.8% to 53.5% [11].

### 1.2 Mindful Eating as a Potential Moderator

Mindful eating has been identified as a potential moderating factor in the relationship between eating attitudes and body dysmorphic concerns [34]. This approach is characterized by heightened attentiveness and intentionality in food choices [35, 36]. Mindful eating encompasses four dimensions [37]: *Focused Eating* measures an individual's ability to stay attentive to food while eating. *Hunger and Satiety Cues* assess the ability to recognize and respond to hunger and fullness. *Eating without Distraction* examines whether a person eats while engaged in other activities. *Eating with Awareness* looks at unconscious eating behaviors [37].

To better understand mindful eating, it is essential to first examine the broader concept of mindfulness, which originates from Buddhist traditions and remains relatively unfamiliar to modern audiences [35, 36].

At its core, mindfulness emphasizes present‐moment awareness, acceptance, and cognitive decentering [35, 36]. Its use in therapy dates back to the 1970s, when the introduction of the Mindfulness‐Based Stress Reduction (MBSR) program, an 8‐week intervention designed to help individuals manage stress and physical illness [38]. Over time, this approach was adapted into Mindfulness‐Based Cognitive Therapy (MBCT), a structured treatment for depression and anxiety, which is now formally recognized and recommended by the UK National Institute for Health and Care Excellence (NICE) [39].

Mindful eating integrates principles of mindfulness into the experience of eating, promoting awareness of thoughts, emotions, bodily sensations, and behaviors related to food. It encourages attentiveness to internal cues like hunger and satiety, minimizes distractions while eating, and promotes a nonjudgmental awareness of the eating process [40]. Key elements of mindful eating englobe recognizing hunger and satiety cues, which help individuals eat in response to physiological needs rather than emotional triggers or external influences. Eating with awareness involves paying close attention to the taste, texture, and enjoyment of food, which can enhance meal satisfaction and reduce overeating. Conversely, eating with distraction, such as watching TV or scrolling on a phone, can lead to mindless consumption, making it harder to regulate portion sizes and recognize feelings of fullness [40]. When individuals lack mindfulness in their eating habits, they may develop inappropriate eating patterns such as binge eating, emotional eating, and external eating, where food consumption is driven by emotions or environmental cues rather than physiological hunger [41, 42].

Research suggests that individuals who engage in mindful eating develop a healthier and more conscious relationship with food, which can help reduce the impact of body image concerns on eating behaviors and attitudes. In fact, beyond its role in regulating food intake, mindful eating also influences body image perceptions and body dysmorphic concerns. Individuals who eat without awareness may be more prone to negative body evaluation due to their reliance on external appearance‐based validation rather than internal bodily cues [25, 43, 44]. By minimizing stress and guilt associated with food choices, mindful eating enhances self‐awareness, enabling individuals to differentiate between physical hunger, satiety, and emotional triggers for eating [44, 45].

The intersection of mindful eating, body dysmorphic concerns, and eating attitudes is particularly relevant for young dietitians, who are frequently exposed to nutritional ideals and esthetic pressures in both academic and professional settings. Mindful eating may act as a moderating factor in this dynamic, enabling dietitians to develop healthier eating attitudes despite body image concerns [46]. By encouraging self‐compassion and self‐awareness, mindful eating can reduce the likelihood of engaging in restrictive, impulsive, or guilt‐driven eating behaviors, promoting a more balanced and intuitive approach to food [40, 45].

### 1.3 The Present Study

The prevalence of body dysmorphic concerns is rapidly increasing, both globally and across the Middle East, reflecting a concern of an urgent need for psychological and behavioral interventions [47, 48]. In Lebanon, this issue is further compounded by persistent political, social, and economic instabilities, which have created a climate of uncertainty, significantly impacting psychological well‐being as was suggested by a recent study conducted by Al‐Khalil et al. [49]. Chronic exposure to financial insecurity, job instability, and socio‐political turmoil has been identified as a key stressor that exacerbates anxiety, stress, and depressive symptoms. These psychological burdens, in turn, are strongly linked to unhealthy eating attitudes, disordered eating behaviors, and body image disturbances [50, 51]. Although direct data on Lebanese dietitians remain limited, they are part of the same socio‐economic context and are therefore not immune to these challenges and may experience similar psychological and dietary struggles. Building on this context, our study aims to explore the moderating effect of mindful eating in the relationship between body dysmorphic concerns and eating attitudes among Lebanese dietitians.

## 2 Methods

### 2.1 Study Design and Participants

A total of 215 young Lebanese dietitians participated in this cross‐sectional study. Participants were aged between 25 and 45 years; however, age was not collected as a variable in the questionnaire and was therefore not included in the statistical analysis. Data were collected through an anonymous and self‐administered online questionnaire, which covered sociodemographic information, body dysmorphic concerns, mindful eating, and eating attitudes. Inclusion criteria required participants to be either practicing dietitians or nutrition students pursuing their degree at the time they filled out the questionnaire. The study utilized a snowball sampling technique, where initial participants were asked to share the questionnaire with their peers and colleagues, using a Google Form link distributed on several social media platforms (Facebook, Instagram, and WhatsApp). This approach facilitated the recruitment of eligible participants, particularly given the relatively specialized population of practicing dietitians and nutrition students.

### 2.2 Ethical Approval

The study protocol was approved by the Holy Spirit University of Kaslik (USEK) Research Ethics Committee—Ethics Certificate number: HCR/EC 2024‐029. The authors assert that all procedures contributing to this work comply with the ethical standards of the relevant national and institutional committees on human experimentation and with the Helsinki Declaration.

### 2.3 Minimal Sample Size Calculation

A minimum sample of 144 participants was deemed enough as per the G*power software Version 3.0.10, using the linear multiple regression: fixed model, *R* ^2^ deviation from zero procedure, with an alpha error of 0.05, a power of 80%, and eight covariates to be entered in the final model. The expected effect size was set at *R* ^2^ = 0.10, which was selected to represent a small‐to‐moderate effect size because no previous studies among Lebanese dietitians were available to provide an empirical estimate for this association. This approach is commonly used when prior evidence is limited; however, it should be acknowledged that interaction effects in moderation analyses are typically smaller than main effects and may require larger samples to be detected reliably. Accordingly, while the present sample exceeded the minimum required for the overall regression model, it may have been less optimally powered for the interaction term specifically.

### 2.4 Questionnaire

The first part of the questionnaire, developed in Arabic, comprised an explanation of the study's topic and objectives. A statement was provided to assure respondents about the anonymity of their participation. Additionally, participants were informed of the importance of providing their informed consent prior to engaging in the study. The second section of the questionnaire encompassed the collection of sociodemographic information from participants, such as sex, residence, marital status, employment, and living situation, as well as self‐reported current weight and height. Subsequently, the BMI was computed according to the World Health Organization (WHO) guidelines [52]. The Household Crowding Index, which serves as an indicator of the family's socioeconomic status, was calculated as the ratio of the total number of individuals residing in the household to the total number of rooms within the dwelling (excluding kitchens and bathrooms) [53]. The physical activity index is determined by the combined result of daily activity intensity, duration, and frequency. It serves as a comprehensive measure to assess individuals' overall physical activity levels [54].

The third part included the following scales:

### 2.5 Eating Attitudes Test (EAT‐7)

The Arabic validated version of the Eating Attitudes Test (EAT‐7) [55] was used to assess eating attitudes. This scale, through its seven items, measures symptoms and concerns characteristic of eating disorders, with responses varying from 0 (never) to 3 (always). For example: “Avoid eating when I am hungry.” Higher scores indicate more inappropriate eating attitudes (Cronbach's *α* = 0.75).

### 2.6 Dysmorphic Concern Questionnaire (DCQ)

The Arabic validated version of the Dysmorphic Concern Questionnaire (DCQ) [56] is a seven‐item self‐report measure designed to assess preoccupation with physical appearance and concerns about perceived bodily imperfections. For example: “Have you ever felt excessively preoccupied with a specific aspect of your appearance?”; “Do you frequently spend time concealing or altering perceived physical flaws?” Each item is rated on a 4‐point scale, where higher scores indicate greater dysmorphic concerns (Cronbach's *α* = 0.85).

### 2.7 Mindful Eating Behavior Scale

The Arabic validated version of the Mindful Eating Behavior Scale (MEBS) was used to assess mindful eating habits [37]. This 17‐item scale measures four key aspects of mindful eating: focused eating, hunger and satiety cues, eating with awareness, and eating without distraction. Each item is rated on a five‐point scale from 1 (never) to 5 (very often), with higher scores indicating greater mindful eating. Since the four aspects are distinct, the scale does not provide a total combined score. The Cronbach's *α* values were as follows: focused eating (0.85), hunger and satiety cues (0.84), eating with awareness (0.77), and eating without distraction (0.68).

### 2.8 Statistical Analysis

Statistical analysis was performed using SPSS software v.25. Internal consistency was assessed by calculating Cronbach's *α* values. The eating attitudes score was deemed normally distributed, as its skewness and kurtosis values fell within the acceptable range of −1 to +1. To compare two means, the Student's *t*‐test was applied, while Pearson's correlation was used to examine relationships between continuous variables. Moderation analysis was conducted using PROCESS Macro (version 3.4, Model 1), an SPSS add‐on. Interaction terms were examined by analyzing the relationship between the predictor and eating attitudes at three levels of the moderator: the mean, 1 standard deviation (SD) below the mean, and 1 SD above the mean. Results of the moderation analysis were adjusted for covariates that showed *p* < 0.25 in the bivariate analysis. *p* < 0.05 was considered statistically significant.

## 3 Results

### 3.1 Sociodemographic and Other Characteristics of the Sample

A total of 215 participants completed the survey, with females representing 97.7% of the sample. Additional descriptive statistics are presented in Table 1.

**Table 1:** Sociodemographic and other characteristics of the sample (*N* = 215).

Variable	*N* (%)
Sex	
Male	5 (2.3%)
Female	209 (97.7%)
Living situation	
Alone	72 (33.8%)
With family	141 (66.2%)
Employment	
No	130 (61.3%)
Yes	82 (38.7%)
Marital status	
Single	148 (69.2%)
Married	66 (30.8%)
Residence	
Urban	123 (57.5%)
Rural	91 (42.5%)

	Mean ± SD
Body mass index (kg/m^2^)	23.78 ± 2.57
Household crowding index (person/room)	1.03 ± 0.57
Physical activity index	28.84 ± 24.91
Eating attitudes	5.39 ± 3.87
Body dysmorphic concerns	7.90 ± 4.48
Focused eating	16.90 ± 4.12
Hunger and satiety cues	16.01 ± 3.81
Eating with awareness	8.61 ± 2.41
Eating without distraction	11.39 ± 2.80

*Note:* Numbers may not sum to the total N due to missing values, which accounted for less than 5% of the dataset.

#### 3.1.1 Bivariate Analysis of Factors Associated With Disordered Eating

A higher mean disordered eating score was found in participants who are employed versus not (Table 2). Moreover, higher body dysmorphic concerns and physical activity were significantly associated with more disordered eating, whereas a higher household crowding index was significantly associated with lower disordered eating (Table 3).

**Table 2:** Bivariate analysis of factors associated with disordered eating.

Variable	Mean ± SD	*t*	*df*	*p*
Sex		1.64	212	0.102
Male	8.20 ± 3.03			
Female	5.33 ± 3.88			
Living situation		0.65	211	0.515
Alone	5.57 ± 4.06			
With family	5.21 ± 3.74			
Employment		−2.18	210	**0.031**
No	4.89 ± 3.48			
Yes	6.13 ± 4.36			
Marital status		1.92	212	0.057
Single	5.69 ± 4.13			
Married	4.70 ± 3.18			
Residence		1.59	212	0.114
Urban	5.74 ± 4.04			
Rural	4.89 ± 3.62			

*Note:* Numbers in bold indicate significant *p* values.

**Table 3:** Correlations of continuous variables with disordered eating.

	1	2	3	4	5	6	7	8
1. Eating attitudes	1							
2. Body dysmorphic concerns	0.24***	1						
3. Mindful eating—Focused eating	0.02	−0.02	1					
4. Mindful eating—Hunger and satiety cues	0.07	−0.01	0.49***	1				
5. Mindful eating—Eating with awareness	0.12	0.24***	0.001	0.14*	1			
6. Mindful eating—Eating without distraction	−0.04	0.24***	0.09	0.22***	0.60***	1		
7. Household crowding index	−0.18**	−0.14*	0.05	0.02	0.23**	0.25***	1	
8. Physical activity	0.23**	0.03	0.09	0.15*	0.02	−0.13	−0.09	1
9. Body mass index	0.03	0.05	−0.27***	−0.30***	0.16*	0.13	0.26	−0.03

* *p* < 0.05;

** *p* < 0.01;

*** *p* < 0.001.

#### 3.1.2 Moderation Analysis

**Figure 1:**
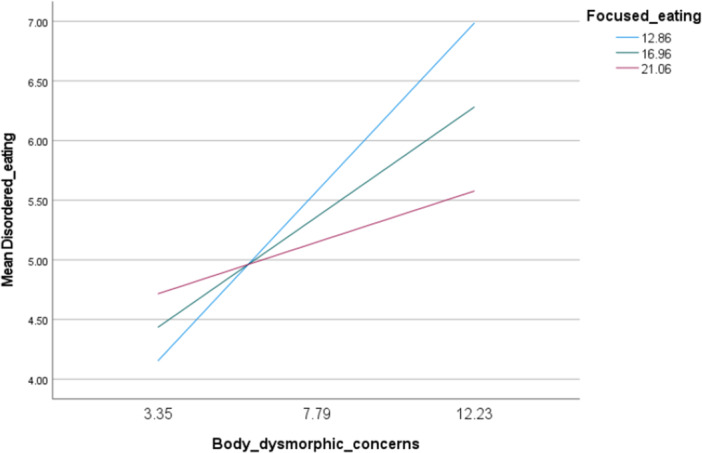
Moderating effect of focused eating between body dysmorphic concerns and disordered eating.

The moderation analysis was adjusted over the following variables: sex, employment status, marital status, residence, household crowding index, and physical activity. The results indicated that focused eating was the only subscale that moderated the association between body dysmorphic concerns and disordered eating (*β* = −0.03, *t* = −2.10, *p* = 0.037, 95%, CI: −0.05; −0.002) (Table 4 and Figure 1). The interaction term explained an additional 1.8% of the variance in inappropriate eating attitudes (∆ *R* ^2^= 0.018, *f*(1,194) = 4.398, *p *= 0.037), indicating a statistically significant but small moderation effect. At low (*β* = 0.32; *p* < 0.001) and moderate (*β* = 0.21; *p* < 0.001) levels of focused eating, higher body dysmorphic concerns were significantly associated with more disordered eating (Table 5).

**Table 4:** Moderating effect of mindful eating between body dysmorphic concerns and inappropriate eating attitudes.

	Unstandardized *β*	Standardized *β*	*t*	*p*	95% CI
Model 1: Focused eating as the moderator (*R* ^2^ = 0.189; ∆ *R* ^2^ = 0.018)					
Body dysmorphic concerns	0.67	0.23	2.96	**0.003**	0.22; 1.11
Focused eating	0.16	−0.04	1.57	0.119	−0.04; 0.36
Interaction of body dysmorphic concerns through focused eating	−0.03	−0.11	−2.10	**0.037**	−0.05; −0.002
Model 2: Hunger and satiety cues as the moderator (*R* ^2^ = 0.176)					
Body dysmorphic concerns	0.36	0.23	1.72	0.087	−0.05; 0.76
Hunger and satiety cues	0.11	0.02	1.09	0.279	−0.09; 0.30
Interaction of body dysmorphic concerns with hunger and satiety cues	−0.01	−0.05	−0.73	0.468	−0.04; 0.02
Model 3: Eating with awareness as the moderator (*R* ^2^ = 0.186)					
Body dysmorphic concerns	0.42	0.20	2.02	**0.045**	0.01; 0.83
Eating with awareness	0.34	0.06	1.84	0.068	−0.03; 0.71
Interaction with body dysmorphic concerns by eating with awareness	−0.03	−0.10	−1.18	0.239	−0.08; 0.02
Model 4: Eating without distraction as the moderator (*R* ^2^ ** = **0.172)					
Body dysmorphic concerns	0.33	0.23	1.44	0.152	−0.12; 0.78
Eating without distraction	0.04	−0.06	0.23	0.818	−0.27; 0.34
Interaction with body dysmorphic concerns by eating without distraction	−0.01	−0.06	−0.52	0.601	−0.05; 0.03

*Note:* Numbers in bold indicate significant *p* values.

**Table 5:** Conditional effects of the focal predictor (body dysmorphic concerns) at values of the moderator (focused eating).

Levels of focused eating	*β*	*t*	*p*	95% CI
Low (= 12.86)	0.32	4.11	**< 0.001**	0.17; 0.47
Moderate (= 16.96)	0.21	3.59	**< 0.001**	0.09; 0.32
High (= 21.06)	0.10	1.22	0.223	−0.06; 0.25

*Note:* Numbers in bold indicate significant *p* values.

In practical terms, this finding suggests that higher levels of focused eating were associated with a weaker relationship between body dysmorphic concerns and inappropriate eating attitudes. Specifically, individuals with higher focused eating showed a less pronounced association between body dysmorphic concerns and inappropriate eating attitudes compared to those with lower focused eating levels.

## 4 Discussion

Eating attitudes and behaviors are influenced by multiple factors, including body image concerns, employment status, physical activity, and environmental stressors [25]. Individuals with body dysmorphic concerns may develop compensatory eating behaviors in an attempt to regulate their appearance, which can potentially lead to disordered eating patterns [57]. However, research linking focused eating, which involves full attention to food consumption and avoidance of distractions, with body satisfaction and eating‐related outcomes remains scarce. This study sought to explore the moderating effect of focused eating on the relationship between body dysmorphic concerns and eating attitudes in a sample of young Lebanese dietitians. To the best of our knowledge, this study is the first to address this topic. Its primary objective is to offer new insights into the psychological dynamics within this group.

### 4.1 Moderating Role of Focused Eating

The most significant and key finding of our study is that one specific dimension of mindful eating, that is, focused eating, plays a moderating role in this relationship, with higher levels of focused eating being associated with a weaker relationship between body dysmorphic concerns and inappropriate eating attitudes. At low and moderate levels of focused eating, higher body dysmorphic concerns were associated with more inappropriate eating attitudes, suggesting that low focused eating may correspond to a stronger association between body dysmorphic concerns and maladaptive eating patterns.

Indeed, prior research suggests that individuals who practice mindfulness and focused eating tend to place less importance on physical appearance, which is associated with a healthier body image and more balanced eating attitudes [58, 59]. In this context, focused eating may be related to a more positive perception of food and body image [59]. By tuning into their meals and internal hunger signals, individuals may lower the perceived influence of societal pressures or unattainable body ideals, which may correspond to a healthier relationship with food and self‐image. In this way, mindful eating, alongside mindfulness techniques, has been associated with more intentional and balanced eating habits that improve both physical and mental well‐being [41].

Therefore, we can infer that practicing focused eating alongside mindfulness techniques may be associated with better stress management and eating‐related outcomes. By promoting a more neutral and less emotionally reactive relationship with food, focused eating may be linked to lower levels of anxiety and maladaptive eating behaviors, although causal conclusions cannot be drawn from the present findings [25, 59, 60].

Lastly, our findings showed that only focused eating moderated the association between body dysmorphic concerns and disordered eating. A possible explanation for the non‐significant effects of the other mindful eating dimensions is that they may capture more general or automatic aspects of eating regulation, which are less directly involved in body image‐driven processes. For instance, sensitivity to hunger and satiety cues primarily reflects interoceptive awareness, which has shown inconsistent associations with body image concerns and may be disrupted in individuals with disordered eating, limiting its moderating role [61, 62]. Similarly, eating without distraction and eating with awareness, while beneficial for overall self‐regulation, may not specifically target appearance‐related cognitions or the evaluative processes underlying body dysmorphic concerns [63]. In contrast, focused eating, by emphasizing deliberate attention to the act of eating, may be more closely related to reduced engagement with maladaptive, appearance‐driven thought patterns [64]. Prior research suggests that different components of mindful eating have distinct psychological correlates and do not uniformly predict eating outcomes, with some dimensions showing weaker or non‐significant associations depending on the population and context [64, 65, 66]. It is worth noting that, although the moderation effect was statistically significant, the additional variance explained by the interaction term was relatively small, suggesting that focused eating may represent only one of several factors associated with the relationship between body dysmorphic concerns and inappropriate eating attitudes.

### 4.2 Clinical Implications

Our findings may help inform awareness initiatives regarding body dysmorphic concerns and disordered eating attitudes among Lebanese dietitians with characteristics similar to those represented in the present sample. At the level of training and professional development, these results may support further exploration of reflection, early identification, and screening approaches in similar population, which may help dietitians better recognize and manage their own attitudes toward food and body image while navigating professional and societal pressures.

While focused eating was associated with a weaker relationship between body dysmorphic concerns and disordered eating attitudes, the cross‐sectional design prevents causal interpretation. Therefore, clinical or educational applications should be considered cautiously pending longitudinal and interventional evidence. At this stage, introducing mindfulness‐informed concepts in educational settings may serve as a preliminary step to enhance self‐awareness and coping, rather than a standardized intervention.

Addressing these concerns may benefit from a multidisciplinary perspective, in which nutritionists, mental health professionals, and educators collaborate to develop approaches that consider both psychological well‐being and dietary behaviors. However, the development of comprehensive or structured programs should be guided by future intervention‐based evidence. Given the strong cultural emphasis on physical appearance in Lebanon, it is crucial to implement culturally sensitive awareness and training approaches that take into account social norms and expectations surrounding food and body image. The current results may inform clinical reflection, but should not yet be interpreted as directly supporting specific therapeutic approaches. For instance, while interventions such as Cognitive‐Behavioral Therapy are commonly used to address distorted beliefs about body image, their application in this specific context would require targeted evaluation.

Finally, the findings of this study emphasize the need for further research into psychological factors, such as self‐esteem, perfectionism, and social comparison, which may influence body dysmorphic concerns and eating attitudes. Longitudinal and interventional studies are particularly needed to determine causal pathways and to inform the development of evidence‐based preventive strategies and interventions.

### 4.3 Limitations

When interpreting the findings of this study, it is important to consider certain limitations. One primary limitation is the cross‐sectional design, which prevents the establishment of causal relationships between variables. The non‐response rate was unknown since the data was collected via a Google Form. Given that the sample was predominantly female, the findings cannot be generalized to male dietitians. Furthermore, the use of non‐probability snowball sampling and social media recruitment may limit the representativeness of the sample and introduce a selection bias. Therefore, the findings should be interpreted as reflecting associations observed within the present sample rather than the broader population of dietitians. Additionally, the data relied on self‐reported questionnaires, which may introduce social desirability bias or inaccuracies in participants' responses. Individuals might have underreported or exaggerated their eating attitudes or body image concerns due to personal perceptions or stigma. Future research could incorporate clinical interviews or objective assessments to enhance data reliability. Another limitation is that age was not collected as a demographic variable and, therefore, could not be included in the analyses. Lastly, future studies should aim to recruit more representative and gender‐balanced samples and employ longitudinal or interventional designs to better clarify the observed associations. Therefore, the findings should be interpreted as reflecting associations observed within the present sample rather than the broader population of dietitians.

## 5 Conclusion

In conclusion, this study suggested that focused eating may play a moderating role in the relationship between body dysmorphic concerns and inappropriate eating attitudes among predominantly female Lebanese dietitians. However, caution is warranted when generalizing these results to the broader population of dietitians, particularly males and those not represented within the sampled social networks. Higher levels of focused eating were associated with a weaker relationship between body dysmorphic concerns and inappropriate eating attitudes. These findings do not imply that focused eating reduces or prevents these outcomes, but rather that it is linked to differences in the strength of their association. Further studies are needed to confirm these findings and explore the broader applicability of focused eating in different populations and contexts, as well as its potential relevance for mental and physical well‐being in healthcare professionals. This study also highlights the need for further research to gain a deeper understanding of the psychological dynamics underlying body dysmorphic concerns, eating attitudes, and eating behaviors in dietitians. Longitudinal studies and experimental designs should be considered to examine causal pathways and temporal relationships, thereby providing deeper insight into the observed moderating role of focused eating.

## Author Contributions

**Elie Ghadban:** writing – original draft. **Joya Sassine:** writing – review and editing. **Sahar Obeid:** supervision. **Lucia Helou:** project administration. **Yonna Sacre:** validation. **Nada Akiki:** project administration. **Feten Fekih‐Romdhane:** validation. **Souheil Hallit:** formal analysis. **Marie Hokayem:** validation.

## Funding

The authors have nothing to report.

## Ethics Statement

All research procedures involving human participants were performed in accordance with the ethical standards of the institutional and/or national research committee and with the 1964 Helsinki Declaration and its later amendments. The study protocol was approved by the Holy Spirit University of Kaslik (USEK) Research Ethics Committee—Ethics Certificate number: HCR/EC 2024‐029.

## Consent

Written informed consent was obtained from all participants prior to their participation. Specifically, consent was considered obtained upon submission of the online questionnaire form, which included a detailed explanation of the study's purpose, procedures, voluntary participation, and confidentiality. Consent was collected by NA and LC between February and May 2024.

## Conflicts of Interest

The authors declare no conflicts of interest.

## Transparency Statement

The lead/Corresponding author, Souheil Hallit, affirms that this manuscript is an honest, accurate, and transparent account of the study being reported; that no important aspects of the study have been omitted; and that any discrepancies from the study as planned (and, if relevant, registered) have been explained.

## Data Availability

The data that support the findings of this study are available from the corresponding author, but restrictions apply to the availability of these data, which were used under license for the current study, and so are not publicly available. Data are, however, available from the authors upon reasonable request and with permission of the ethics committee.
